# The Role of FNDC4 in Inflammation and Metabolism for Various Diseases

**DOI:** 10.14336/AD.2024.0381

**Published:** 2025-05-04

**Authors:** Yu-Xin Hu, Can Hu, Min Hu, Yi-Peng Gao, Wen-Sheng Dong, Kang Li, Yun-Jia Ye, Xin Zhang

**Affiliations:** ^1^Department of Geriatrics, Renmin Hospital of Wuhan University, Hubei Key Laboratory of Metabolic and Chronic Diseases, Wuhan 430060, China.; ^2^Department of Ultrasound, Union Hospital, Tongji Medical College, Huazhong University of Science and Technology, Clinical Research Center for Medical Imaging in Hubei Province, Hubei Province Key Laboratory of Molecular Imaging, Wuhan 430022, China.; ^3^Department of Cardiology, Renmin Hospital of Wuhan University, Hubei Key Laboratory of Metabolic and Chronic Diseases, Wuhan 430060, China.

**Keywords:** FNDC4, biological function, inflammation, metabolize, tumor

## Abstract

Fibronectin (FN) can bind to certain integrin receptors on the cell surface through short peptide sequences, thereby transmitting extracellular stimuli to intracellular effector molecules. FNDC4 plays a similar role due to the constitution of a type III FN domain, which is a binding site for DNA, heparin, or cell surface. It mainly functions as a signal transmitter after being cleaved and secreted as the extracellular N-terminal fibronectin type III domain (sFNDC4). Emerging studies have shown that FNDC4 plays crucial roles in numerous diseases and holds significant implications for guiding clinical treatment. This review aims to summarize the different roles and the latest advances of FNDC4 in the development of various diseases, in order to provide new ideas for clinical treatment.

## Introduction

The fibronectin type III domain containing protein 4 (FNDC4) was first discovered and named by Teufel et al. in 2002 [1]. In 2016, Bosma et al. first discovered that [2] FNDC4 can be cleaved and secreted as the extracellular N-terminal fibronectin type III domain (sFNDC4), which plays an anti-inflammatory role in inflammatory bowel disease (IBD) by affecting macrophage phagocytosis, survival, cytokine secretion, and cell polarization.

Recent studies have shown that FNDC4 plays multiple biological effects in regulating inflammation, glycolipid metabolism, tumors, bone metabolism and skeletal muscle differentiation. Wuensch et al. demonstrated that FNDC4 expression was dramatically elevated in the mucosal samples obtained from patients with colorectal cancer (CRC), indicating an involvement of FNDC4 in the pathogenesis of tumor progression [3]. The expression level of FNDC4 varies in different tumors, indicating a certain relationship with tumor prognosis [4]. Other studies have reported that the RGD sequence of FNDC4 is associated with integrin β1 (ITGβ1) to activate focal adhesion kinase (FAK) and promotes the migration of skeletal muscle derived cells [5]. Besides, FDNC4 can influence the differentiation of C2C12myoblasts by activating Wnt/β-catenin signal transduction [6]. FNDC4 can also act on human visceral adipocytes as an extracellular factor to reduce fat generation and browning [7]. The latest research showed that FNDC4 dramatically reduced the incidence of rheumatoid arthritis (RA) [8], affected the progression of endothelial corneal malnutrition [9], and also contributed to the onset of male schizophrenia [10].

Our unpublished research demonstrated that FNDC4 overexpression of sFNDC4 supplementation protected against aging- or ischemia/reperfusion (I/R)-induced cardiac injury and dysfunction. Mechanistically, we found that FNDC4 restored AMPKa/PPARa-dependeant mitochondrial function, thereby improving lipid metabolism, inflammation and oxidative stress within the aging hearts. Regarding I/R injury, FNDC4 inhibited the proteasomal degradation of HIF1a, reinstated HIF1a protein expression, and subsequently reduced cardiomyocyte apoptosis. Additionally, it facilitated the secretion of FGF1 from cardiomyocytes by activating HIF1a manner, and eventually promoted the proliferation and angiogenesis of endothelial cells in a paracrine manner.

Furthermore, it was mentioned in Mikaël M Martino's study that [11] FNDC4 potentially exerted its effects indirectly by binding to various growth factors, akin to the interaction observed with fibronectin (FN). This interaction is believed to be significant in embryonic development and tissue repair. Consequently, it can be inferred that the matrix-binding variants of FN type III domains 12-14 could serve as a versatile binding anchor for growth factors in tissue engineering and regenerative medicine matrices. This inference provides novel concepts for targeted FN treatment in clinical settings.

**Figure 1.**
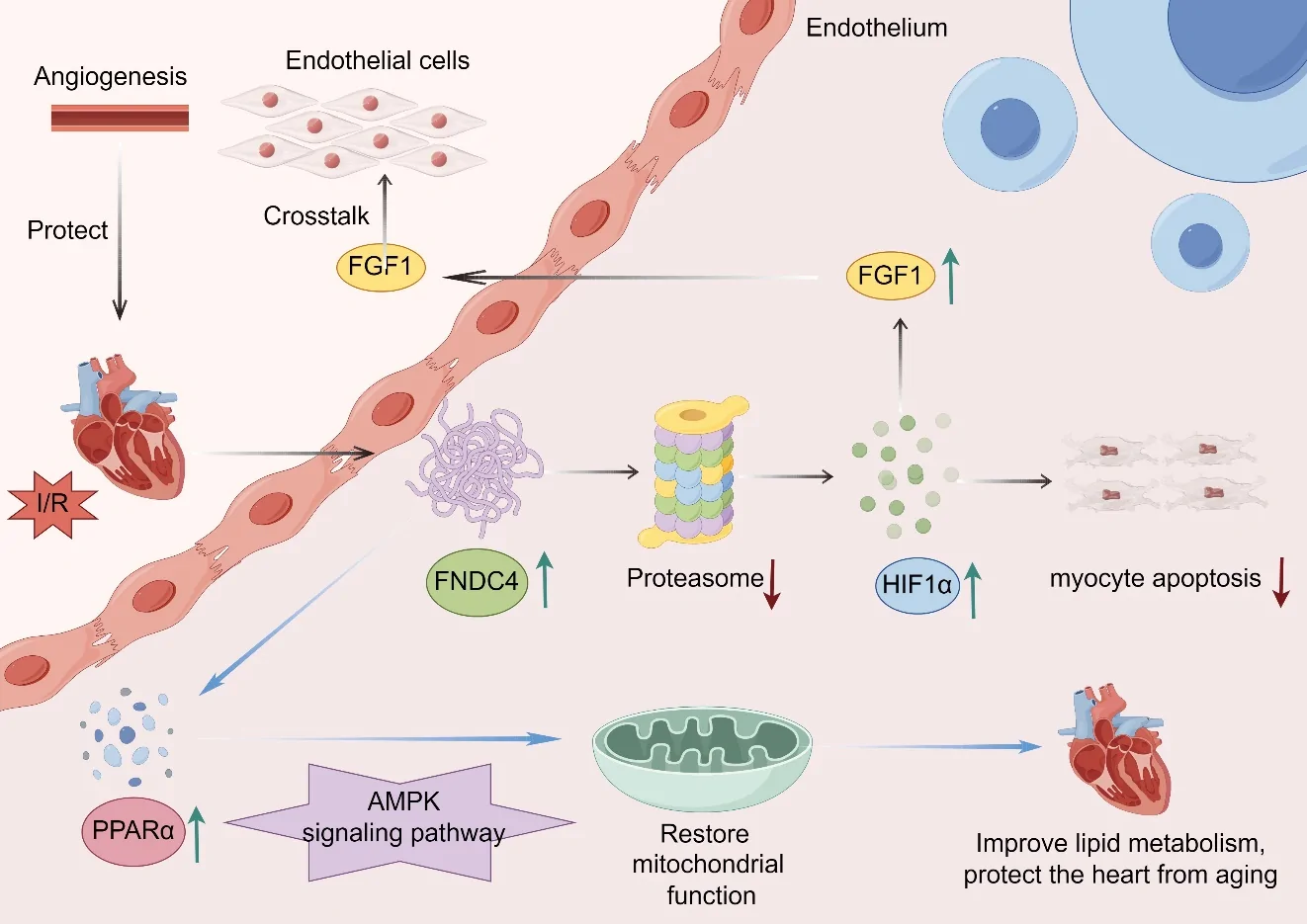
Our unpublished research shows the role of FNDC4 in the heart.

Considering the multifaceted biological effects and wide-ranging roles of FNDC4, we aim to provide a comprehensive review of the localization, receptors, expression patterns, and molecular mechanisms of FNDC4 in various diseases (Fig. 1).

## FNDC family

FN is one of the important components of the extracellular matrix (ECM), and it represents a class of macromolecular glycoproteins that can bind to certain integrin receptors on the cell surface through short peptide sequences [12-14], thereby transmitting extracellular stimuli to intracellular effector molecules [15].

From the UniProt database, we can learn that the protein family of FNDC, characterized by the presence of type III FN domain, contains a total of 11 human proteins [4], namely FNDC1, FNDC3A, FNDC3B, FNDC4, FNDC5, FNDC6 interleukin-20 receptor subunit (IL20RB), FNDC7, FNDC8, FNDC9, FNDC10, and FNDC11. This family is highly conserved in structure, consisting of signal peptides, type III FN domain, C-terminal intracellular domain, and hydrophobic transmembrane domain [2]. They are implicated in the progression of tissue development, cell adhesion, migration, proliferation and many other biological progresses [3, 16].

FNDC5 is the most studied one among all FNDC proteins, and it plays critical roles in various pathophysiological diseases, including cardiovascular diseases. We recently found that FNDC5 overexpression could inhibit inflammation and oxidative damage, thereby preventing doxorubicin- or aging-related cardiac injury [17-20].

Among all FNDC proteins, FNDC4 and FNDC5 have the strongest homology, with 57% amino acid homology in functional and extracellular domains, and human FNDC4 has 100% homology with mouse FNDC4 [1, 2]. The extracellular domain of FNDC4 can be cleaved, exhibiting the characteristics of extracellular molecules [2], and can transmit extracellular stimuli to corresponding effector molecules within the cell, leading to the latter exerting biological effects within the cell.

Furthermore, the examination of expression and functional characteristics of the FNDC1, FNDC3A, FNDC3B, FNDC4, FNDC5, and FNDC6 genes has unveiled that FNDC1 functions as a regulatory factor in cardiovascular function. It is implicated in hypoxia-induced myocardial cell apoptosis and is also linked to an elevated risk of arterial hypertension [4]. Other studies have shown that FNDC3B can regulate adipogenesis and osteoblast differentiation [21], and glucocorticoids are involved in regulating the expression of FNDC5 [22].

## Location and receptors

**Figure 2.**
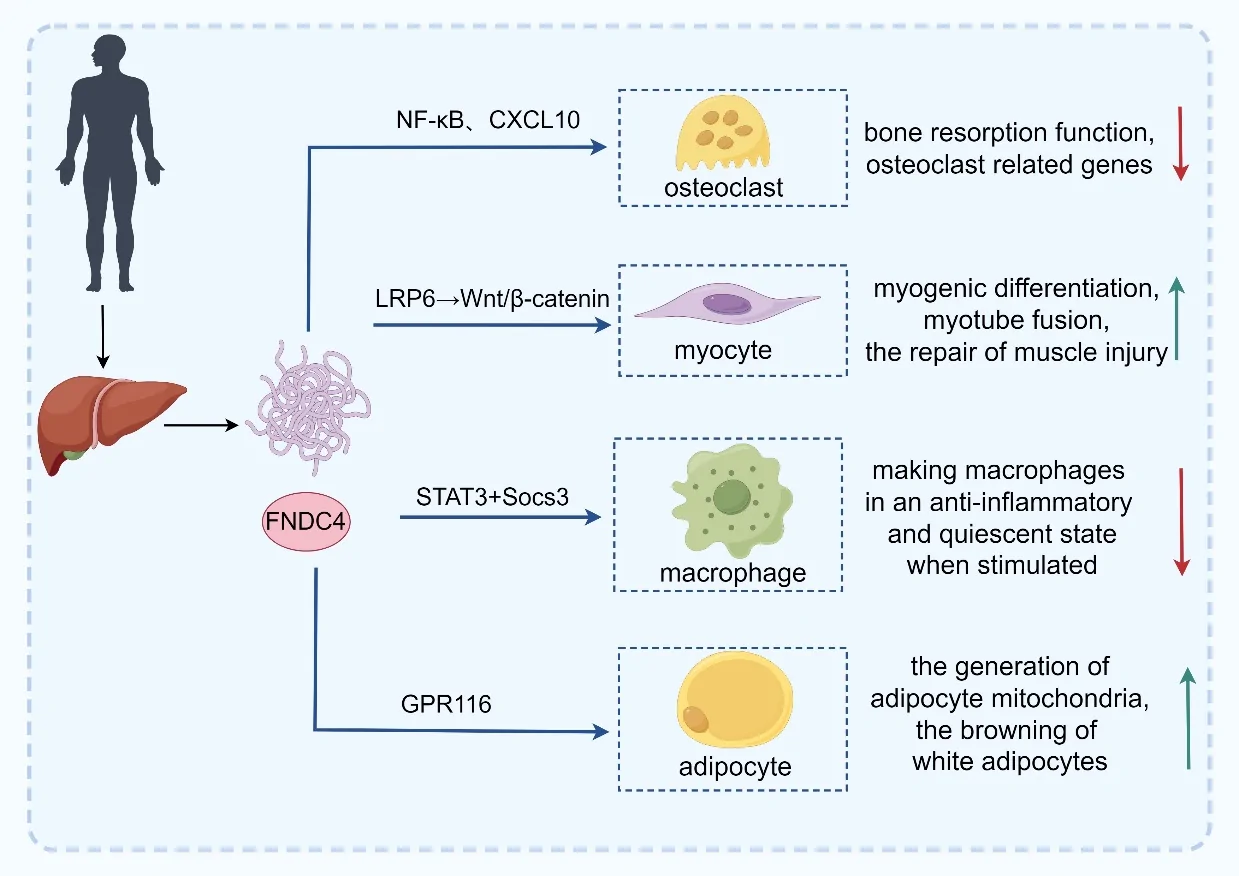
The liver secretes FNDC4 and it plays roles in various tissue cells.

Research has indicated that FNDC4 exhibits the highest expression level in the liver and brain, while its expression in several other tissues such as the heart, skeletal muscle, and adipose tissue is relatively low. However, FNDC4 is barely expressed in macrophages [2]. Anastasia Georgiadi et al. confirmed that [23]the decrease in human liver FNDC4 transcription levels leads to a corresponding decrease in circulating sFNDC4 levels in mice, indicating that the liver is the main source of sFNDC4 (Fig. 2).

There are several conclusions about the receptors and expression patterns of FNDC4. Gema Frühbeck et al. have shown that [7] FNDC4 inhibits adipogenesis in vitro and promotes fat browning in human visceral adipocytes through its receptor ADGRF5 (also known as GPR116). GRP116 is the receptor of sFNDC4 in white adipose tissue, which can promote insulin signaling and insulin mediated glucose uptake in white adipocytes [23], and this receptor is also widely expressed in various tissues such as ovaries [24]. In addition, in bovine bone marrow mesenchymal stem cells, FNDC4 promotes the differentiation and migration of bovine myeloid-derived suppressor cells (MDSCs) through ITGβ1 receptor-mediated FAK signaling pathway [5].

Several studies have also validated that FNDC4 activates the GRP116 receptor, resulting in the phosphorylation of AMP-activated protein kinase (AMPK) and the expression of heme oxygenase-1 (HO-1) in adipocytes [3], thereby inhibiting inflammation and endoplasmic reticulum (ER) stress [25]. Bosma's research has shown that [2] FNDC4 is tightly bound to macrophages and monocytes. Treatment with FNDC4 can inhibit macrophage activity, suppress inflammatory responses, and mediate the activation of an inflammatory regulatory factor STAT3. In the study of mouse skeletal muscle cells, it was confirmed that FDNC4 could activate Wnt/β-catenin signal transduction, and subsequently affected the differentiation of C2C12 cells [6].

## FNDC4 and inflammatory bowel disease

The excessive activity and dysregulation of immune function in the gut dominated by macrophages may lead to the occurrence of IBD. In situ RNA hybridization experiments showed that FNDC4 was upregulated in the inflammatory site of human IBD and positively correlated with inflammatory markers. In the mouse model of dextran sodium sulfate (DSS)-induced colitis, it was discovered that FNDC4-deficient mice exhibited heightened severity of colitis [2], while injection of recombinant FNDC4 protein significantly improved intestinal inflammation, indicating that FNDC4 can reduce the severity of inflammation and disease.

Bosma et al. further clarified that [2] the impact of FNDC4 on macrophages can be partially mediated by a transcription factor STAT3 involved in regulating inflammatory processes and cell survival [26, 27]. STAT3 binds to the promoter region of suppressors of cytokine signaling 3 (Socs3), promoting the upregulation of Socs3, thereby regulating macrophage function [2].

Macrophages can be polarized to M1 or M2 phenotype, however, Bosma's research data showed that [2] FNDC4 did not affect the polarization of macrophage, but resulted in an anti-inflammatory and quiescent-state macrophage.

Previous studies have demonstrated that steroid-refractory acute graft-versus-host disease (GVHD) and inflammatory bowel disease (IBD) share similarities in specific aspects, such as clinical manifestations, pathogenic mechanisms, and genetic risk factors [28]. The International IBD Genetics Consortium (IIBDGC) has compiled a comprehensive list of gene loci and identified 26 HLA alleles independently associated with IBD, including statistically significant variants associated with IBD [29-31]. Nevertheless, the precise extent of the association between these mutations and the risk of graft-versus-host disease (GVHD) following allogeneic hematopoietic cell transplantation (HCT) remains uncertain. Therefore, Paul J. Martin conducted a genome-wide association study (GWAS) in a cohort of 1980 HCT recipients of European ancestry and HLA matched relatives or non-relatives donors [32], and used the same method to test the association between HLA alleles independently associated with IBD and stage 2 to 4 gastrointestinal GVHD. The results showed that among the 296 single nucleotide polymorphisms (SNPs) and 26 HLA alleles detected, rs1260326 in SNPs was associated with Crohn's disease and various other phenotypes. Its C allele was associated with increased expression of FNDC4 in the transverse colon, but not in the terminal ileum or sigmoid colon. Speculation suggests that the C allele of these single nucleotide polymorphisms (SNPs) may be associated with elevated expression of FNDC4 in stimulated intestinal epithelial cells. However, it is important to note that this study exclusively included recipients of European ancestry, which imposed certain limitations.

Based on the above research findings, we can speculate that FNDC4 and its association with macrophages may be an attractive therapeutic target for the treatment of IBD and intestinal GVHD.

## FNDC4 and bone or muscle

As is well known, maintaining a balance between osteoblast-mediated bone formation and osteoclast-mediated bone destruction is essential for a stable bone metabolism [33]. Excessive osteoclast activity may cause excessive bone destruction and lead to many osteolytic diseases [34, 35].

The directed differentiation of osteoclasts is regulated by two important cytokines [36, 37], among which the receptor activator of nuclear kappa-B ligand (RANKL) can stimulate the directed differentiation of osteoclast precursor cells into osteoclasts by binding to its receptor RANK and the activation of the downstream nuclear factor κB (NF-κB) signaling pathway [38-40].

Lyu's research suggests that [41] FNDC4 could inhibit multiple functions of osteoclasts through dose-dependently suppressing NF-κB. FNDC4 at 1000ng/mL could greatly weaken the bone resorption function of osteoclasts. In an independent study, it was verified that FNDC4 inhibited osteoclast differentiation by downregulating an important regulatory protein CXCL10 [42, 43], and the addition of CXCL10 flips the inhibition of FNDC4 on osteoclasts through its receptor CXCR3. In addition, FNDC4 can inhibit the upregulation of RANKL-mediated CtsK, TRAP, and downstream target gene nuclear factor of activated T cells cytoplasmic 1 (NFATc1), indicating that FNDC4 can inhibit the expression of osteoclast related genes. Thus, FNDC4, acting as a regulatory factor in osteoclast differentiation, holds promising implications for the treatment of diverse osteolytic diseases.

Satellite cells (MDSCs) derived from bovine muscle generally remain inactive, but can differentiate into skeletal muscle cells under certain conditions [44, 45]. Wang et al. conducted high-throughput sequencing analysis and observed a significant increase in the expression of FNDC4 during the differentiation process of bovine MDSCs. They further confirmed that FNDC4 is involved in ITGβ1 binding and regulates the differentiation and migration of bovine MDSCs through FAK [46].

Li et al. demonstrated through Western blot experiments that [6] during the differentiation process of C2C12 muscle cells, the content of FNDC4 and the marker of muscle cell differentiation myogenin (MYOG) gradually increase, indicating that FNDC4 promotes the differentiation and generation of muscle cells. Among signaling proteins, the Wnt family members play an important role in skeletal muscle differentiation [47-51], and FNDC4 can activate the classic Wnt/ β-catenin signaling pathway by phosphorylating low-density lipoprotein related receptor 6 (LRP6) to promote β-catenin expression and nuclear translocation, and then promote myogenic differentiation, myotube fusion, and participate in the repair of muscle injury [6].

The above experimental studies indicate that FNDC4 can serve as a novel target, providing new ideas for the clinical treatment of bone and muscle diseases.

## FNDC4 and tumor

**Figure 3.**
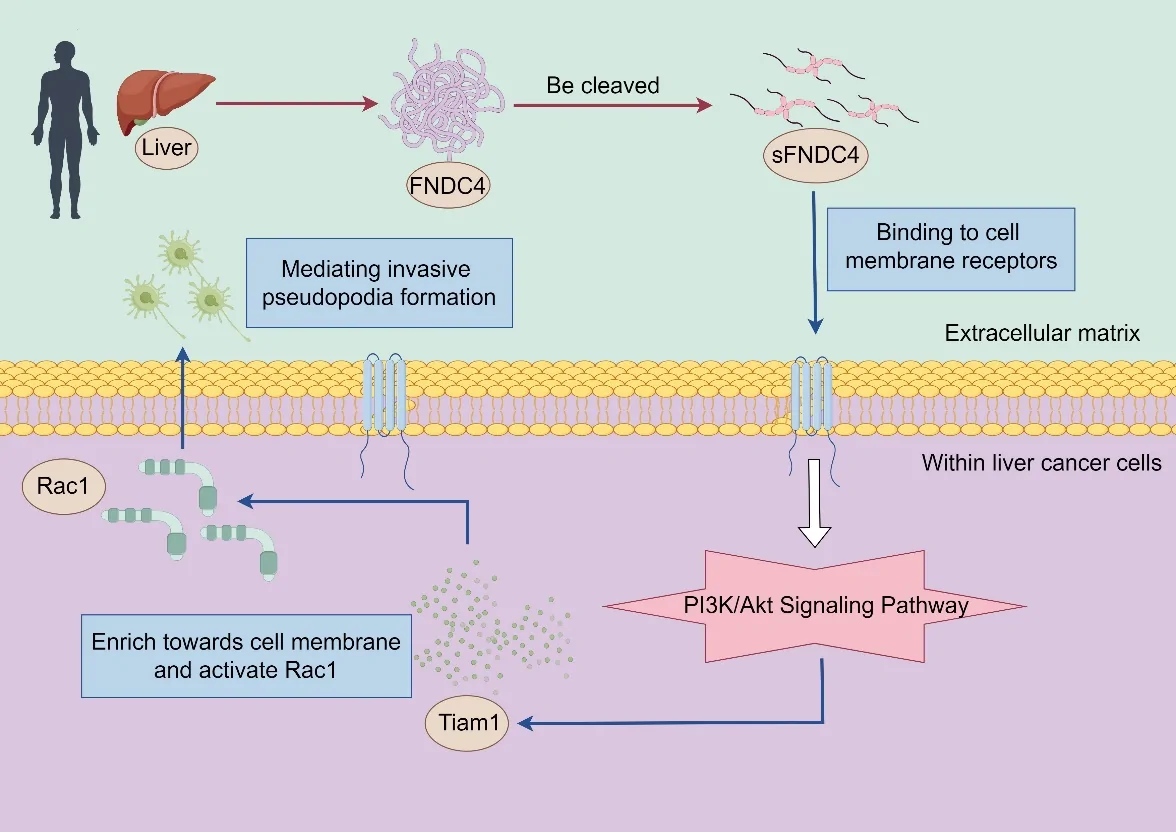
FNDC4 activates the PI3K/Akt signaling pathway and ultimately enhance HCC invasion and metastasis.

Cancer is the second most common cause of death. Jiang et al. found in their study on the expression and prognostic analysis of the fibronectin type III domain protein family in human cancer [4] that the mRNA expression levels of FNDC1, FNDC3A, and FNDC3B were significantly higher in most malignant tumors than in nearby normal tissues, while the expression levels of FNDC4, FNDC5, FNDC6, and FNDC8 were significantly reduced. Specifically, the expression level of FNDC4 in invasive ductal carcinoma of breast (IDC), lobular and ductal mixed breast cancer, liver cancer (HCC), serous adenocarcinoma of ovary, esophageal cancer, prostate cancer, sarcoma, etc. is low. The research results indicate that members of this family can serve as new prognostic biomarkers or potential targets for human cancer treatment and have significant research significance (Fig. 3).

One of the important mechanisms in the process of tumor cell invasion and metastasis is the formation of pseudopodia mediated by local aggregation of Rac1 near the cell membrane [52], and the activity of Rac1 can be regulated by T-lymphoma invasion and metastasis gene 1 (Tiam1) [53]. The localization of Tiam1 cell membrane is a key link determining Tiam1/Rac1 activity, which is regulated by the PI3K/Akt signaling pathway [54, 55]. Based on the above background, Wang et al. proposed and confirmed that [56] FNDC4 activates the PI3K/Akt signaling pathway by acting on cell surface receptors, inducing Tiam1 enrichment towards liver cancer cell membranes and activating Rac1, thereby promoting Rac1 mediated invasive pseudopodia formation and ultimately enhancing HCC invasion and metastasis. At the same time, through clinical feature analysis of 205 HCC patients and survival analysis based on FNDC4, it was concluded that high expression of FNDC4 is positively correlated with microvascular invasion and low differentiation of liver cancer cells. Moreover, the group with high expression of FNDC4 has a shorter overall postoperative survival and a higher recurrence rate, indicating a close correlation between high levels of FNDC4 and poor prognosis of hepatocellular carcinoma, Li et al.'s experiment also has the same conclusion [57]. Their experiment provided a more in-depth supplementary explanation of the role of FNDC4 in HCC, that is, the expression level of FNDC4 in HCC is lower than that in normal tissues. If the content of FNDC4 increases, it may indicate a poor prognosis for patients.

Bladder cancer (BLCA) is one of the most common malignant tumors of the urinary system. Zhao et al. [58] constructed a new necrosis related gene (NRG) signature containing FNDC4 to predict the survival of the TCGA-BLCA cohort, and validated the accuracy of the NRG score using an external dataset. Their results indicate that the NRG score has good performance in evaluating the prognosis and treatment sensitivity of BLCA patients and can guide clinical treatment.

In recent years, the incidence rate of thyroid cancer has risen rapidly, and FNA cytopathology is the most accurate method for preoperative diagnosis. Wang et al. [59] developed a specific splicing variant that combines multiple genes, including FNDC4, in order to better characterize thyroid nodules using technically challenging but clinically relevant diagnostic FNA samples. However, we still need further validation trials to develop it, and then to guide preoperative surgical decisions for thyroid tumors.

Glioblastoma is one of the most malignant tumors in the central nervous system because of its strong invasiveness and poor prognosis. Li et al.'s study found [60] that the expression of FNDC4 is associated with poor prognosis, and exogenous FNDC4 inhibits M1 polarization of M0 macrophages without M2 polarization; This situation was also observed in glioblastoma cells overexpressing FNDC4. This indicates that FNDC4 can guide the evaluation of the prognosis of glioblastoma.

Radiochemotherapy is one of the treatment options for patients with advanced head and neck squamous cell carcinoma (HNSCC) [61]. However, at the same time, different proportions of patients may experience local or distant recurrence. The use of biomarkers can indicate which patients have a higher risk of recurrence. In a study by Mercedes Camacho [62], the transcriptional expression of FNCF4 is significantly associated with disease-specific survival in HNSCC patients receiving radiotherapy and chemotherapy. The disease-specific survival rate of patients with elevated FNDC4 expression is significantly reduced, indicating that patients with elevated FNDC4 have a higher possibility of HNSCC recurrence.

It should be noted that previous studies have shown that FNDC4 is upregulated in IBD. However, Wuensch T's study confirmed that FNDC4 and its receptor GPR116 did not show significant changes in colorectal cancer [3]. This still requires more comprehensive experimental research to verify.

In summary, variations in the expression levels of FNDC4 across different tumors have been observed in various research findings, necessitating the verification through repeated experiments or larger sample sizes. It can be speculated that FNDC4 might serve as a potential therapeutic target or prognostic marker for several types of cancer. However, it is crucial to analyze different tumors individually, as this approach holds positive implications for guiding the clinical treatment of tumors.

## FNDC4 and obesity

Obesity is a risk factor for type 2 diabetes mellitus (T2D), nonalcoholic fatty liver disease and cardiovascular disease, which is closely related to systemic chronic inflammation, insulin resistance and other pathological conditions [63, 64]. At present, studies have found that FNDC4 directly participates in lipid metabolism of adipocytes, thereby affecting the pathological and physiological states related to obesity [7, 23, 25]. Gema Frühbeck et al.'s study showed that [7] plasma FNDC4 was reduced in morbidly obese patients, and their visceral adipose tissue (AT) showed high expression of FNDC4 and its receptor GPR116, which is not related to the degree of insulin resistance. The content of FNDC4 is regulated by adipogenesis, lipolysis, and pro-inflammatory stimulation in human visceral adipocytes, and can reduce intracellular lipid accumulation.

AT plays an important role in energy balance and homeostasis. White adipose tissue mainly stores energy and undergoes browning to transform into brown adipose tissue under specific stimuli, shifting energy balance from storage to consumption. This mechanism may provide new strategies for obesity treatment [65]. Gema Frühbeck et al. found that [7] FNDC4 intervention in human adipocytes promotes the expression of the adaptive thermogenic marker UCP1 protein and upregulates the transcription levels of brown and brown adipocyte markers; Simultaneously, it has been observed that the expression of FNDC4 in the visceral adipose tissue of obese individuals exhibits a negative correlation with the expression of the macrophage biomarker CD68. This finding further supports the notion that FNDC4 plays an anti-inflammatory role in obese individuals. After intervention with FNDC4, the mitochondrial DNA content in human adipocytes increased by about 2 times, indicating that FNDC4 can promote the generation of adipocyte mitochondria and the browning of white adipocytes, thereby participating in the body's energy metabolism.

More than that, Gema Frühbeck also conducted a study on the potential role of obesity in increasing susceptibility to COVID-19 complications in obese patients [66]. And it was found that, AT, as the host and target organ of SARS-CoV-2 virus, is overactivated in obesity, inducing cell apoptosis. The potential beneficial effects of FNDC4 on COVID-19 outcomes include anti-obesity [2, 7, 67] and anti-inflammatory [2, 68] activities, such as inhibiting M1 polarization of macrophages and pro-inflammatory cytokine production in AT. Their research suggests that FNDC4 may lead to a better prognosis in obese patients with COVID-19.

Overall, research has shown that FNDC4 levels are closely related to obesity related factors such as body fat, weight, and systemic inflammatory status, and may become a new therapeutic target for improving obesity related metabolic disorders.

**Figure 4.**
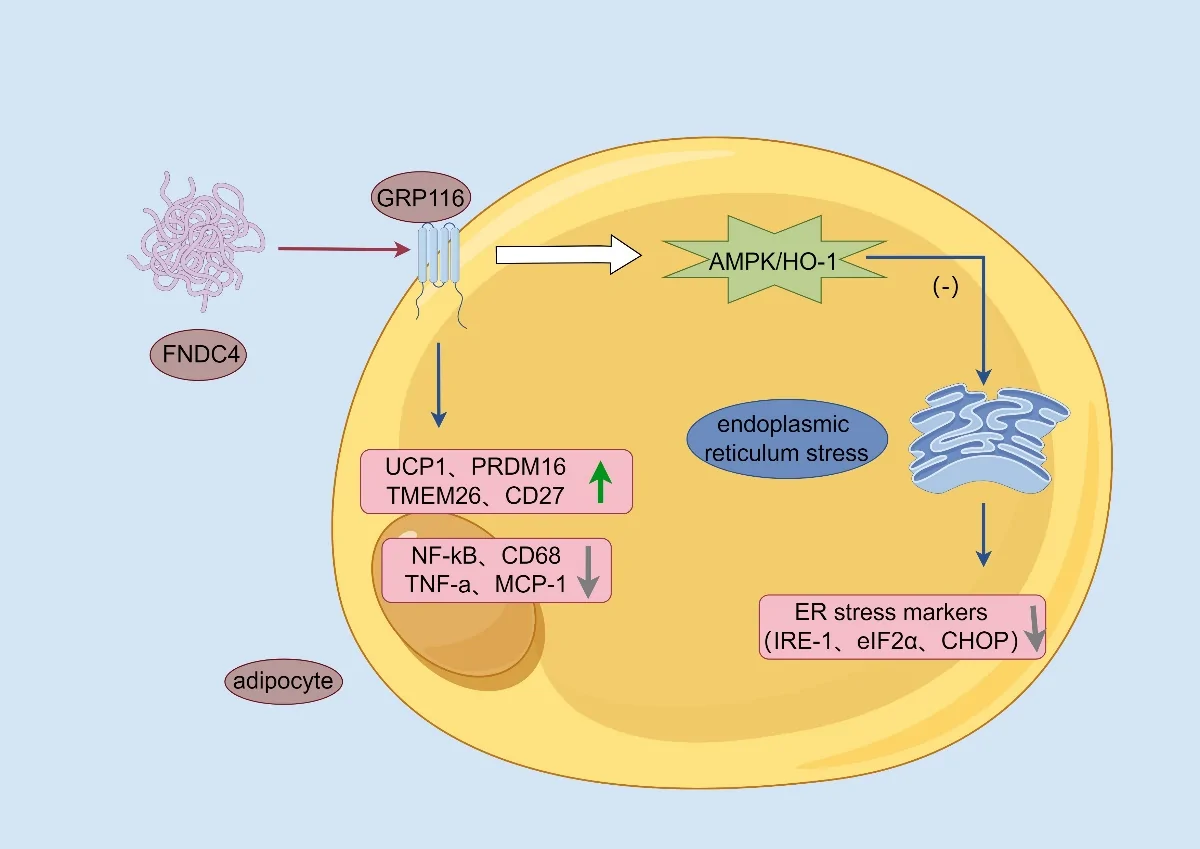
FNDC4 can improve insulin resistance by inhibiting inflammation and ER stress through the AMPK/HO-1 mediated pathway.

## FNDC4 and type 2 diabetes mellitus

The removal of excess glucose from blood circulation is central to the pathogenesis of obesity related T2D. It is known that insulin resistance in AT is related to glucose transport, lipid uptake, and fat breakdown, among which chronic inflammation is closely related to insulin resistance in adipose tissue [69]. Activated macrophages in adipose tissue recruit and release cytokines that promote fat breakdown, such as TNF-α, IL-6 and other factors can lead to insulin resistance in AT. Previous studies have shown that FNDC4 participates in the body's glucose metabolism by improving insulin resistance [70]. Anastasia Georgiadi found that GPR116 is a receptor for sFNDC4 in white adipose tissue, and glucose homeostasis is controlled by FNDC4-GPR116, the liver-white adipose tissue endocrine axis [23]. The liver mainly controls the circulating level of sFNDC4, and the reduction of liver factor FNDC4 leads to pre-diabetes in mice, that’s because sFNDC4 promoted insulin signal transduction and insulin mediated glucose uptake in white adipocytes. Nie et al found that [71], mice with GPR116 selectively knocked out in AT were more susceptible to impaired glucose tolerance and insulin resistance induced by high-fat diet, and showed increased levels of circulating triacylglycerol, increased ectopic lipids in liver and skeletal muscle, and increased levels of inflammatory factors, suggesting that the FNDC4-GPR116 axis is closely related to systemic insulin resistance. Georgiadi et al also found that [23], supplementation of FcsFNDC4 in prediabetic mice improved glucose tolerance and inflammatory markers in a white-adipocyte selective and GPR116-dependent manner. This discovery opens up the possibility of utilizing this endocrine circuit as an alternative therapeutic strategy for prediabetes associated with obesity. However, the weight of liver, muscle, adipose tissue and triacylglycerol content of high-fat fed mice after long-term injection of FNDC4 did not change significantly, indicating that the change of lipid content in metabolic tissue cannot fully explain the improvement of glucose tolerance by FNDC4.

In addition, multiple studies have shown a close relationship between ER stress and insulin resistance. Elevated ER stress was detected in the adipose tissue of obese subjects with insulin resistance [72]. Suzuki et al. reported that adipose ER stress plays a crucial role in the development of insulin resistance and glucose intolerance [73]. Based on the above background, Wonjae Lee conducted a study on the effects of FNDC4 on adipocyte ER stress, inflammation, and insulin resistance mediated by hyperlipidemia [25]. Their results show that FNDC4 can improve insulin resistance by inhibiting inflammation and ER stress through the AMPK/HO-1 mediated pathway, suggesting that FNDC4 may be a new therapeutic agent for insulin resistance and T2D (Fig. 4).

In conclusion, FNDC4 may offer a novel treatment approach for diabetes associated with obesity. Additionally, targeting and modulating endoplasmic reticulum stress in adipose tissue could prove to be an effective strategy for combating insulin resistance. However, further exploration is needed to determine whether FNDC4 directly participates in glycogen breakdown, gluconeogenesis, and other glucose metabolism, in order to gain a deeper understanding of the specific mechanisms by which FNDC4 affects glucose metabolism.

## FNDC4 and female reproduction

The fertility of women mainly depends on their nutritional status. As is well known, obesity can reduce a woman's fertility, while having too little body fat can also lead to amenorrhea, anovulation, or pregnancy failure [74].

Previous studies have shown that FNDC4 can cleave and release a bioactive protein sFNDC4 that can regulate insulin sensitivity in peripheral tissues. The receptor for this protein, ADGRF5, is expressed in the ovaries [24]. Mathilde Daudon's research has shown that [75] cows require more energy to produce milk during lactation than energy intake [76, 77], resulting in a negative energy balance (NEB) that drives the mobilization of body fat and even muscle tissue. Sudden mobilization of adipose tissue increases plasma FNDC4 concentration, which may improve glucose tolerance in the reproductive tract, but its impact on ovarian function remains to be determined. The interplay between FNDC4 and other adipokine concentrations potentially influences how the reproductive system perceives energy status and contributes to fertility. These studies may suggest novel approaches to mitigate postpartum infertility.

Premature menopause or primary ovarian insufficiency (POI) refers to menopause before the age of 40 [78]. A cross-sectional study by Mohammad Reza Mirinezhad [79] compared 117 premature menopausal women with 183 healthy women, and the results showed that one of the SNPs associated with premature menopause was rs2303369, and this variant gene can encode FNDC4. These studies suggest that FNDC4 may also be associated with premature menopause in women.

## Conclusion and Prospect

FNDC4, as a novel liver factor, plays a positive biological role in regulating inflammation, bone and muscle differentiation, and glucose and lipid metabolism. Its concentration changes may reflect the prognosis of different tumors, and it is expected to become a biomarker and therapeutic target for new metabolic diseases. Our team's unpublished research has also shown that FNDC4 can improve lipid metabolism and protect against cardiac aging and promote FGF1 secretion in cardiomyocytes through HIF1a dependent manner, thereby promoting endothelial cell proliferation and angiogenesis, thereby protecting against myocardial I/R injury. These studies suggest that FNDC4 may play a certain role in cardiac aging and metabolism.

Meanwhile, FNDC4 has 100% homology between mice and humans, further increasing the possibility of clinical translation based on mouse research results. For example, we can speculate and experimentally verify that supplementing with exogenous rFNDC protein can improve intestinal inflammation in IBD and have therapeutic effects on aging hearts; On the contrary, inhibiting FNDC4 through targeted drugs may inhibit the invasion and metastasis of HCC; In addition, FNDC4 can promote the generation of adipocyte mitochondria and browning of white adipocytes, inhibit inflammation and ER stress to improve insulin resistance. Intervention with FNDC4 in obese patients with insulin resistance may have therapeutic effects.

Nevertheless, despite the progress made in understanding the role of FNDC4 in various systemic diseases, there remain numerous unresolved issues that require further clarification. Firstly, regarding the changes in the expression level of FNDC4 in different tumors, there are differences in the research results, and it is necessary to analyze different tumors separately, which still needs to be verified through repeated experiments or expanding sample sizes. Secondly, the precise mechanism through which FNDC4 exerts its effects on different tumor cells has not yet been fully elucidated. Finally, in relation to the influence of FNDC4 on female reproduction, existing research still possesses limitations and lacks in-depth exploration. Further research and improvements are necessary to enhance our understanding in this area. Consequently, translating these fundamental findings into clinical treatments remains a significant challenge.
